# Adapting and pre-testing the World Health Organization’s Caregiver Skills Training programme for autism and other developmental disorders in a very low-resource setting: Findings from Ethiopia

**DOI:** 10.1177/1362361319848532

**Published:** 2019-05-16

**Authors:** Bethlehem Tekola, Fikirte Girma, Mersha Kinfe, Rehana Abdurahman, Markos Tesfaye, Zemi Yenus, WHO CST Team, Erica Salomone, Laura Pacione, Abebaw Fekadu, Chiara Servili, Charlotte Hanlon, Rosa A Hoekstra

**Affiliations:** 1 King’s College London, UK; 2 Addis Ababa University, Ethiopia; 3 Yekatit 12 Hospital Medical College, Ethiopia; 4 St. Paul’s Hospital Millennium Medical College, Ethiopia; 5 Joy Center for Children with Autism, Ethiopia; 6 World Health Organization, Switzerland; 7 University of Milan-Bicocca, Italy

**Keywords:** caregivers, developmental delay, developmental disorders, Ethiopia, parent-mediated, parenting skills programme, qualitative

## Abstract

The World Health Organization’s Caregiver Skills Training programme for children with developmental disorders or delays teaches caregivers strategies to help them support their child’s development. Ethiopia has a severe lack of services for children with developmental disorders or delays. This study explored the perspectives of Ethiopian caregivers, professionals and other stakeholders to inform adaptation and implementation of the World Health Organization’s Caregiver Skills Training in Ethiopia. Data collection included (1) a consultation and review, comprising stakeholder meetings, review of draft Caregiver Skills Training materials and feedback from Ethiopian Master Trainees and (2) a pre-pilot including quantitative feasibility and acceptability measures and qualitative interviews with caregivers (n = 9) and programme facilitators/observers (n = 5). The consultation participants indicated that the Caregiver Skills Training addresses an urgent need and is relevant to the Ethiopian context. Several adaptations were proposed, including more emphasis on psycho-education, stigma, parental feelings of guilt and expectations of a cure. The adapted Caregiver Skills Training was pre-piloted with excellent participation (100%) and retention (90%) rates. Four themes were developed from the qualitative data: (1) Programme acceptability and relevance, (2) Perceived programme benefits, (3) Challenges and barriers and (4) Suggestions for improvement. The World Health Organization’s Caregiver Skills Training addresses a local need and, with careful adaptations, is feasible and acceptable to be implemented in Ethiopia. These findings may have relevance to low-resource settings worldwide.

Ethiopia is a low-income African country of over 104 million people (United Nations, Department of Economic and Social Affairs, Population Division, 2017). Like most African countries, Ethiopia has a severe shortage in mental health care provision (Fekadu & Thornicroft, 2014). One of the major barriers to scaling up the service provision for children with autism or other developmental disorders or delays (DD) and their families is the lack of skilled human resources (Patel, Kieling, Maulik, & Divan, 2013). In Ethiopia, this lack is particularly pronounced, with currently only one formally qualified child psychiatrist working in the country and 20 clinical psychologists, none of whom are specialised in child mental health. Services for children with DD are mainly restricted to the country’s capital city, Addis Ababa, and are therefore inaccessible to the majority (85%) of the population living in rural areas (Tekola et al., 2016; Zeleke, Hughes, & Chitiyo, 2018). Interviews with caregivers of a child with DD who had successfully found their way to a child mental health clinic in the capital indicated that 75% were in need of appropriate education for their child, and 47% expressed a need for medical support (Tilahun et al., 2016). Most children with DD in Ethiopia receive little or no formal help and there is a very limited access to structured, evidence-based psychosocial interventions for affected families.

Several systematic reviews of studies, mostly conducted in high-income settings, suggest that caregivers are able to learn the skills necessary to deliver intervention strategies to their children with DD (McConachie & Diggle, 2007; Odom, Boyd, Hall, & Hume, 2010; Reichow, Servili, Yasamy, Barbui, & Saxena, 2013). These reviews also indicate that children, as well as families, benefit from these interventions, even if they are short and of low intensity. Reviewing the available evidence up to 2012 from studies mostly done in high-income settings, Oono, Honey, and McConachie (2013) reported strong evidence for improved parent–child interaction and parent synchrony and some evidence for improved language comprehension and reduction of autism severity. Since then a small number of studies have explored the effects of parent-mediated interventions in middle-income settings India and Pakistan (Juneja et al., 2012; Rahman et al., 2016 and Nair et al., 2014). These studies reported a reduction in autism severity (Juneja et al., 2012; Nair et al., 2014), improved child expressive language (Juneja et al., 2012), increased child social and language skills (Nair et al., 2014) and improved spontaneous child communication with the parent (Rahman et al., 2016). While these studies addressed some of the issues pertinent in low-resource settings, the interventions were still delivered by specialists in two of these studies (Juneja et al., 2012; Nair et al., 2014) and interventions were conducted with individual caregivers, not in a group. In low-resource settings such as Ethiopia, community-based psychosocial interventions delivered by non-specialists are a viable strategy to scale-up support for families with children with DD (Divan et al., 2015; Patel et al., 2013) and scalability is more likely when the intervention is group-based rather than delivered to caregivers individually.

The World Health Organization (WHO) and international partners recently collaborated in the development of an evidence-based Caregiver Skills Training (CST) programme for families of children with autism and other DD. The WHO CST was developed to be low in intensity, of relatively short duration and deliverable by non-specialist providers to groups of caregivers, thus meeting the affordability and feasibility criteria of low-resource settings (Reichow et al., 2014; Reichow et al., 2013). The CST aims to teach caregivers strategies to engage their child in communication and play and promote adaptive behaviours and learning as well as reduce challenging behaviour (Salomone et al., 2018).

Its content is based on principles of social learning theory, positive parenting, applied behaviour analysis and developmental theories. The programme consists of a combination of nine group sessions for caregivers and three individual home visits. In the nine group sessions, each has a specific focus: (1) introduction and psycho-education, (2) engaging with the child, (3) helping children share engagement, (4) understanding communication, (5) promoting communication, (6) preventing challenging behaviour, (7) responding to challenging behaviour, (8) learning new skills and (9) problem solving and self-care. The group sessions use a range of learning techniques including modelling, role-play, demonstrations, group discussions and case vignettes. The first home visit (prior to the CST start) is used to define specific goals and targets for each family, explore the presence of additional health problems, and inform and engage other caregivers; the two further home visits (midway and at the end of the training) focus on coaching the caregiver and providing tailored support, evaluating progress, trouble shooting and identifying possible additional support needs.

Data on the effectiveness of interventions for children with autism or other DD outside high-income country settings are extremely limited, with studies so far focusing on middle rather than low-income countries (Hamdani et al., 2017; Hastings, Robertson, & Yasamy, 2012; Rahman et al., 2016). Although the WHO CST package is designed to be used in low-resource settings, it has never been implemented in very low-income contexts such as Ethiopia. Apart from a lack of resources and services, Ethiopia’s context is characterised by high levels of stigma (Tekola et al., 2016; Tilahun et al., 2016; Tilahun et al., 2017), very low levels of awareness of autism and other DD (Tekola et al., 2016) and misconceptions about the causes of DD (Tekola et al., 2016; Tilahun et al., 2016). There is thus a need to explore whether the WHO CST programme meets the local needs, whether the programme required any adaptation to local context and whether it is acceptable and feasible to implement the programme in the Ethiopian context.

This study aimed to explore the perspectives and experiences of Ethiopian caregivers, professionals and other stakeholders on the WHO CST programme to inform its adaptation and implementation in the Ethiopian culture and context. The study comprised two phases: (1) a consultation and review phase that informed the adaptations of the programme and (2) a pre-pilot conducted in a clinical setting to explore the acceptability and feasibility of the programme.

## Methods

This study is part of an ongoing project aiming to test the adaptability, feasibility and acceptability of the WHO CST programme in urban and rural Ethiopian settings. The study used a combination of data collection methods: (1) consultation and review comprising interactive stakeholder meetings, a detailed review of draft CST materials, and feedback from Ethiopian Master Trainees, (2) quantitative data collected prior to and during the CST pre-pilot and (3) qualitative interviews with caregivers and programme facilitators/observers who took part in the pre-pilot of CST. By involving multiple stakeholders in our consultation and review and using multiple data collection methods, we were able to triangulate information gained regarding the relevance of the WHO CST programme for the Ethiopian setting and the adaptations needed to suit the local context. Based on this study phase, the programme was adapted and subsequently pre-piloted. The quantitative and qualitative data collected in the pre-pilot were used to evaluate the feasibility and acceptability of the programme. Acceptability was measured quantitatively by consent rate to be included in the CST programme, retention rate during group sessions and home visits, and through participant feedback sheets completed following each home visit. Acceptability was assessed qualitatively through in-depth interviews. Feasibility was assessed quantitatively through timing each CST group session and qualitatively through in-depth interviews. The study was approved by the Institutional Review Board of the College of Health Sciences of Addis Ababa University (#062/16/Psy) and the Psychiatry, Nursing and Midwifery subcommittee of King’s College London’s College Research Ethics Committee (#RESCM-17/18-3489).

### Consultation and review

#### Stakeholder meetings

In April 2015, July 2015, August 2016 and August 2017, interactive workshops were organised in Addis Ababa to solicit stakeholders’ views on local training needs, CST content, CST delivery considerations and implementation of the CST in urban versus rural Ethiopian settings. The stakeholders included representatives of the Ministry of Health, from three Ethiopian centres for autism and intellectual disabilities (some of whom are parents of children with DD), three local non-governmental organisations working with children with DD, three psychiatrists and psychologists and two researchers with expertise in community-based rehabilitation programmes. Detailed notes were taken during each meeting, including the programme changes suggested; a report based on these notes was sent to all participants shortly afterwards for their approval and further feedback.

#### Review of draft WHO CST materials

In August 2016, all draft CST materials (‘WHO CST field test version 1.0’), comprising facilitator manuals and guides and participant booklets, were reviewed in detail by seven reviewers (authors F.G., M.T., Z.Y., C.H., R.A.H. and two additional expert reviewers). Potential cultural issues, contextual and methodological considerations and issues of feasibility and acceptability were noted, and revisions were suggested.

#### Feedback from Ethiopian Master Trainees

During a Training of Trainers workshop in May 2017, feedback on CST content, length and intensity was received from Ethiopian Master Trainees. A pool of seven Master Trainers (three general psychiatrists, one MSc clinical psychologist, two BSc psychologists and one diploma-level community rehabilitation worker) were trained in the CST programme during an intensive 5-day course in Addis Ababa, facilitated by a WHO CST team member (L.P.) and a CST specialist working for Autism Speaks. Throughout the course, detailed notes were taken regarding concepts or activities that trainees found difficult to understand, or where trainees suggested revisions to make the programme easier to understand or more culturally or contextually appropriate.

### CST pre-pilot

The adapted CST programme was pre-piloted in the child mental health clinic at Yekatit 12 Hospital in Addis Ababa, led by a specialist (M.T. or R.A.) and assisted by two non-specialist facilitators. While the CST is ultimately designed to be delivered by non-specialists in a community setting, the programme was tested first in a clinical setting facilitated by specialists to allow for expert response should challenges emerge. We therefore consider this a ‘pre-pilot’ rather than a true pilot, given that the programme was delivered by a specialist facilitator. Observers were a clinical psychologist or a psychiatrist (R.A., when not facilitating sessions herself) at Yekatit 12 Hospital.

Ten caregivers who had a long-term caring responsibility for a child aged 2–9 years with a DD and living within easy travelling distance of the training site were invited to take part in the pre-pilot. Caregivers were approached through Yekatit 12 Hospital and schools for children with DD in Addis Ababa.

### Quantitative data collected prior to and during the CST pre-pilot

Before the pre-pilot started, caregiver-reported demographic and service history information were collected from all participating caregivers and their children. Throughout the programme, participation and retention rates during group sessions and home visits were collected, as well as the time it took to complete each CST group session. Brief feedback was also solicited regarding the caregivers’ acceptability of the home visits.

### Qualitative interviews with caregivers and programme facilitators/observers who took part in the CST pre-pilot

After the pre-pilot was completed, qualitative interviews were conducted with the CST facilitators leading or assisting the CST sessions (n = 3), CST observers (n = 2) and each of the participating caregivers (n = 9). The aims of these interviews were to explore (1) the experiences of facilitators and observers who provided the first delivery of CST and caregivers’ experience participating in the CST, (2) the caregivers’ experience working with the concepts and strategies presented in the programme, (3) the acceptability and appropriateness of the programme within the local context and (4) key informants’ suggested revisions to the programme and suggestions for future training, supervision and mentoring of CST facilitators. The interviews were guided by topic guides (see Supplementary materials) and were conducted in Amharic by B.T., who was independent from the team delivering the CST. All interviews were audio tape-recorded and lasted 45–60 min.

### Data analysis

B.T. read and re-read the reports from all consultation and review meetings before extracting major points. The quantitative data collected during the CST pre-pilot were analysed descriptively to assess acceptability (indicated by participation and retention rates) and feasibility of the programme (indicated by duration of each CST group session). All qualitative interviews were transcribed verbatim and translated into English by researchers from Addis Ababa University who are bilingual in Amharic and English. B.T. checked the accuracy of transcripts by comparing them with audio recorded interviews. Inductive thematic analysis was used to analyse interview transcripts (Braun & Clarke, 2006; Clarke & Braun, 2013). First, all interview transcripts were repeatedly read to become familiar with the data and make notes of early impressions. Next, B.T. and R.A.H. independently generated initial codes for three interview transcripts and iteratively agreed on a final code book after B.T. had coded all transcripts. Data were coded using NVivo 11 software. All codes were then organised into initial themes and sub-themes. B.T. and R.A.H. met again to review and modify the preliminary themes and sub-themes by assessing the data associated with each theme. Finally, themes and sub-themes were refined and rich data extracts that illustrate each of these themes were selected.

This study used triangulation to ensure a more comprehensive and reliable understanding of the adaptability, feasibility and acceptability of the CST programme. Denzin (1978) and Patton (1999) described four kinds of triangulation: (1) method triangulation (using multiple methods of data collection about the same phenomenon), (2) investigator triangulation (using multiple investigators in the same study), (3) theory triangulation, (using multiple theories to analyse and interpret data) and (4) data source triangulation (using multiple sources of data). In this study, we used three of these: (1) multiple sources of data (we consulted and interviewed local stakeholders, professionals and caregivers), (2) multiple investigators (during thematic analysis of the qualitative data, B.T. and R.A.H. independently generated initial codes and iteratively agreed on a final code book) and (3) multiple methods of data collection (we used consultation meetings, document review, feedback from trainees, quantitative feasibility and acceptability measures and qualitative interviews).

## Results

### Consultation and review findings

The participants in the consultation and review exercise made several recommendations on the WHO CST version 1.0 materials, including feedback regarding the length, content, complexity and cultural appropriateness of the teaching materials and home visits (see Table 1). Collating data from the first three consultation meetings and Master Training (i.e. based on all feedback up to May 2017) in Ethiopia, with data from consultation meetings, Master Trainings and pilot testing of the programme in other countries participating in the global CST field testing initiative, the WHO CST Team produced a revised version 2.0 of the programme materials for piloting. The Ethiopian team further adapted these revised materials to address the remaining comments raised in the consultation and review phase (see Table 1). All changes were made and recorded in Word using track changes. Final adapted materials were translated to Amharic by bilingual English-Amharic speakers with expertise in mental health. Prior to commencing the translation, the translation of key CST terms such as ‘caregiver’, ‘developmental disorder’, ‘pretend play’ and ‘shared engagement’ was agreed following discussion in the stakeholder consultation meeting in August 2017, ensuring each translated term was easy to understand and respectful to families, while preserving the original meaning. All translated materials were checked and harmonised by F.G. and by a psychiatrist with no prior CST experience.

**Table 1.** Recommendations and adaptations made to the WHO CST programme for implementation in Ethiopia.

Ethiopia review of WHO CST materials field test version 1.0			Revised WHO CST materials, field test version 2.0	Ethiopia adaptation of WHO CST materials field test version 2.0
Methods	Topic	Comment	Revision	Cultural adaptation
Stakeholder meetings; Expert review of CST draft materials	Length and complexity of sessions	Length of group sessions differs; some sessions are very long (impractical for caregivers given the need to arrange childcare); some sessions contain materials that are too complex	Sessions each containing 2 h of content	Adapted the programme to be suitable for non-literate populations by removing any need for written taught delivery (e.g. using a white board) instead replacing it with oral discussions (suiting the Ethiopian oral tradition) and simplifying the participant booklets, removing lengthy written texts
Stakeholder meetings	Acceptability home visits	Home visits are acceptable as long as it is made clear in advance that facilitators will not accept any food or drinks, to ensure family resources are not compromised		Stakeholders’ suggestion implemented
Expert review of CST draft materials	Inclusion of picture schedule as communication strategy	Use of picture schedule is counter-intuitive to many caregivers in Ethiopia; one school for children with autism tried to implement it without success. In contrast, gestures are commonly used to visualise actions		Replaced picture schedule with gestures
Stakeholder meetings	The appropriateness of cross-cultural illustrations provided by WHO	Considered culturally appropriate by local stakeholders		None
Stakeholder meetings	The names of the people represented in cross-cultural case narratives	The names should be changed to local Ethiopian names		Changed the names of the people represented in case narratives to local Ethiopian names
Stakeholder meetings; Feedback from Ethiopian Master Trainees	Additional topics to include	There is a need for greater emphasis on psycho-education, stigma, parental feelings of guilt and expectations of a cure	Stronger emphasis is given to psycho-education, addressing beliefs about causes, parental guilt and stigma, initial session 1 split into 2, so total programme became 9 rather than initial 8 sessions	Expectation of cure explicitly addressed in programme information sheet and group session 1
		Helpful strategies to discipline a child (avoiding physical punishment) should be includedTraining should more strongly highlight the active role caregivers can play to support their child’s development; particularly, the role of play in interaction between caregiver and child	Stronger emphasis is given to how parents can support their child’s development, particularly through the inclusion of case vignettes	Physical punishment explicitly addressed in additional discussion activity in group session 6Importance of play with caregivers explicitly addressed in additional discussion activity in group session 3

WHO: World Health Organization; CST: Caregiver Skills Training.

### Quantitative findings pre-pilot

#### Response, engagement and retention

All invited caregivers (N = 10; 100%) consented to participate in the pre-pilot, and 9 out of 10 caregivers (90%) engaged with the programme. One caregiver who consented could not get leave from work to attend the sessions and therefore did not engage with the programme. All nine caregivers who enrolled in the programme continued participation until completion. Demographic characteristics of the participating families are summarised in Table 2. Seven of the participating caregivers were mothers; contrary to our expectations (in Ethiopia, childcare responsibilities are traditionally reserved for women), two participants were fathers.

**Table 2.** Health and demographic information of caregivers and children participating in the pre-pilot.

Caregivers	Main diagnosis of child	Child’s age (years) and gender	Caregiver’s age (years) and parental role	Caregiver’s educational level	Caregiver’s employment and living arrangement
C1	Autism	4; boy	41; father	12th grade*	Employed; lives with wife & three children
C2	Intellectual disability	9; boy	35; mother	5th grade	Employed; lives with one child
C3	Intellectual disability	7; boy	37; mother	2nd grade	Employed; lives with husband & one child
C4	Intellectual disability	7; boy	40; mother	No formal education	No paid job; lives with husband & four children
C5	Autism	9; boy	43; mother	No formal education	No paid job; lives with husband & four children
C6	Intellectual disability	8; girl	43; father	12th grade + 3 years further education	Employed; lives with wife & two children
C7	Intellectual disability	6; girl	39; mother	Basic literacy	Employed; lives with one child
C8	Autism	5; boy	30; mother	12th grade	No paid job; lives with parents & two children
C9	Intellectual disability	7; girl	42; mother	11th grade	No paid job; lives with one child

* Completion of 12th grade is equivalent to completion of high school.

While the CST sessions were planned to last 2–2.5 h, in practice each session took around 3 h to complete, including a break. At each session, between two and six caregivers brought their child because they could not find childcare; in response, we organised childcare at the CST programme site from the second session onwards. Feedback from home visits suggested that these were highly acceptable; all caregivers indicated that the home visits were ‘very useful’ or ‘useful’; six out of nine thought the sessions were the right length; one found the session too short and two found the session too long.

### Findings from the qualitative interviews

Four themes were developed using thematic analysis in relation to the CST. Two additional themes relating to the challenges of raising a child with DD and protective resources will be discussed in a separate article. The four themes relating to the CST were (1) Acceptability and relevance of the programme; (2) Perceived benefits of the programme; (3) Challenges in relation to participating in, delivering and implementing the programme and (4) Suggestions for improvement (see Table 3). Each of the four themes is presented below and illustrated with representative quotes. Full quotes from all participants relating to each theme and sub-theme can be found in the Supplementary materials (matrices 1–4). Caregiver informants are referred to as C1, C2, C3 and so on; Programme facilitators and observers are referred to as FO1, FO2, FO3 and so on.

**Table 3.** List of themes and sub-themes that were developed using thematic analysis.

Themes and sub-themes
1. Acceptability and relevance of the programme
2. Perceived benefits of the programme
2.1. Improved knowledge and skills
2.2. Positive effects on psychological wellbeing
2.3. Changed perception
2.4. Sharing experiences with other caregivers
3. Challenges and barriers
3.1. Participant challenges
3.1.1. Practical
3.1.2. Socio-cultural
3.2. Programme delivery challenges
3.2.1. Practical
3.2.2. Other
4. Suggestions for improvement

### Acceptability and relevance of the programme

Generally, interviewees’ comments regarding the acceptability and relevance of the programme were positive. All caregivers described the content of the programme as very useful. Although initially some caregivers expressed scepticism about its relevance to their situation, they indicated that after they took part in the training, they found it to be reflective of their feelings and experiences:

C8: … I didn’t expect that they [the CST team] will know what we were feeling deep down and they would present it to us in the lessons. I didn’t know that they knew [what we were feeling inside] … I came here just to check it out … when I took one class, I saw a lot of the things that were in my life. All the problems I had at home were written down in this book [participant booklet] …

Programme facilitators and observers put forward that caregivers’ acceptance of the programme was also expressed in their eagerness to attend the training. They noted the high attendance rates and that all caregivers arrived on time:

FO1: one other thing I have observed is that all parents, as far as I know, have attended all the sessions … When one parent could not come, he sent his wife to attend the training … this shows how they have taken the programme seriously and believe that it is very useful for them.

Caregivers also stressed their keenness to attend and how much they enjoyed it:

C8: … I was happy to come [to the training] leaving everything behind. There is nothing more important for me than this issue.

C2: … we had a very good time. Nine weeks felt like a week …

### Perceived benefits of the programme

#### Improved knowledge and skills

Many of the interview participants reported that the training improved caregivers’ knowledge and skills with regard to how they can promote their child’s independent life skills and communication:

C4: … previously my son did not dress himself … now he pushes one of his arms through one sleeve and I help him with the other sleeve. He puts on his shoes by himself and I tell him to put them on the right feet … it [the training] is very useful.

C5: since I understand now how to help him say words, thank God now my child starts to say words. He is asking what he wants … when he wants *injera* [Ethiopian flatbread] he says, ‘give me *injera’* and when he wants water, he says ‘give me water’. This is a big change and I am very happy.

Many caregivers reported that the training helped them to understand and manage their child’s challenging behaviour:

C8: … because I didn’t understand my child’s behaviour, I used to think that he was spoilt, or I was a weak mom. When I get angry with his behaviour, I used to think that it is because I am a bad mom …. when he does something wrong now, I understand that he doesn’t do it intentionally.

Some caregivers noted that after they took part in the training, they changed how they discipline their child, leading to an improved relationship with their child:

C8: … I used to hit him a lot so that he keeps quiet …. but after I took the training, I have not raised my hands to him.

C5: Before I took part in the training, as I told you, I used to yell at him [my child] and as a result he feared me, and he only approached his sisters. He was also scared of his dad as he shouted at him even more than I did … the training was useful for me because my child is now closer to me than before … a child needs love …

Caregivers indicated that the training helped them to recognise the importance of self-care. For example, one mother noted that she used to neglect herself and that the training taught her the importance of looking after herself so that she can look after her child.

#### Positive effects on psychological wellbeing

Many caregivers, particularly mothers, noted that the training helped them to manage their stress and to assist their child in their day-to-day life:

C3: The training was very useful for me. I didn’t know how to handle my child before this training. I always felt stressed. He didn’t know how to put on his clothes quickly but [each morning] I ordered him to put on his clothes quickly because if it is 8:30am I may be late for my work … even when we walked on the street, I usually told him to walk fast … I used to get stressed and made him stressed. Now this lesson has helped me a lot. I now know lots of things. Now I calm myself down and help him to get dressed. It has helped me a lot.

In addition to the knowledge and skills they gained from the training, one mother indicated that meeting up with other parents helped her to manage her stress. Another mother said that the training helped her to have social life which brought about positive effect on her emotional wellbeing:

C4: … Now I am mixing up with people. Previously I did not mix up with people. I did not mix up with anybody. I used to lock my door and stay at home and only open the door when my children come home from school. I am good now because they [CST facilitators] are encouraging me when they come for the home visit …

#### Changed perception

Many caregivers suggested that the training changed their perspective on their child’s development:

C2: … [After we took part in the training] we have changed a lot … eh … I was hopeless … eh I used to think that my child can’t mix with other people. Now my child has come out and I also came out, many people have seen us. We met with many parents. We shared with each other our pains and many other things and I believe we are going to bring about many changes …

C9: especially for me it has given me strength … eh … it made me to think that I have hope …

A CST facilitator and observer provided similar comments:

FO4: … I have been here for all the nine weeks, in this small period the results are the changes in the attitude of the caregivers. They started to think of changing their environment for the comfort of their children … at the end, what I have observed is courage. Courage which says even if you [CST facilitators] are not going to be with us all the time I will walk through life with my child.

FO1: apart from acquiring skills, it [the training] also helped them to correct the negative attitude they get from different sources. To give you an example, every one’s children can grow, every child can develop. This is very useful information because, for most of them, traditionally or medically when they are told that the diagnosis is developmental disorder, [….] they don’t have an expectation that their child can develop further. They have an attitude that it is impossible for the children to improve and make changes. Therefore, it was very useful and positive in this respect.

#### Sharing experiences with other caregivers

Many caregivers talked about the benefits of sharing experiences with other caregivers:

C7: … I received many lessons from the parents. We have learned from each other’s experiences…

C8: … It’s a big deal to be able to share with people about your experience. When you can share the things that you didn’t even share with your family you become very happy…

However, one of the fathers considered sharing experiences with other caregivers as a barrier. He said listening to other people’s experiences of the home practice took a long time and as a result may have affected what the caregivers were able to learn from the training. He also emphasised that not all children with DD behave and act in the same way, so listening to other people’s experiences may not always be helpful. His perspectives may also be related to him feeling uncomfortable sharing experiences in a predominantly female group.

### Challenges and barriers

The key informants highlighted several challenges and barriers in relation to participating in, delivering and implementing the programme. The challenges could be grouped into two sub-themes: (1) Participant challenges and (2) Programme delivery challenges.

#### Participant challenges

This sub-theme captures the challenges caregivers faced in participating in and implementing the training programme, including practical issues and social and cultural barriers.

##### Practical

Many caregivers talked about the difficulty of finding childcare, indicating that apart from their close relatives, no one understands their child’s behaviour. In the absence of childcare, several caregivers brought their child with them to the training, and some reflected on the difficulties this caused:

C6: It is difficult to bring your child. I have brought mine once to the training. The training took a long time. Naturally such kind of children get bored quickly and feel agitated.

Some of the caregivers indicated that finding transport was a major issue:

C1: transport is one of the problems … after we stayed here up to 4pm I found it difficult to get transport to go home. I arrived home late at night. If my [spouse] was not there to look after my child who would look after him? That is very difficult.

Many caregivers also talked about the challenge of implementing what they have learnt in the programme at home with their child. Lack of time and work burden were the major obstacles identified:

C1: I have not implemented all of what I have learnt at home. That is due to lack of time. I have three children.

C3: I have work burden. I wake up in the morning and take him [my child] to school. Then at 3pm I go to his school straight from my workplace … to bring him home from school. When I arrive at home, I need to do all the household chores … I become very tired …

##### Socio-cultural

Some caregivers and programme observers talked about lack of support from the caregivers’ family to attend the training:

FO4: One thing I have observed is that one of the caregivers’ husband said to his wife ‘do not waste your time on something you are not going to bring about change’. She was arguing with him very much saying that what about the benefits I get from it [the training] …

C3: I have one uncle. He said it [the training] won’t change your life why don’t you leave it ….

#### Programme delivery challenges

Caregivers, facilitators and observers also reported challenges in relation to delivering the training.

##### Practical: lack of preparation

Programme facilitators and observers indicated that due to a delay in receiving the translated training materials they were not able to get well-prepared before they delivered the training. This problem was also raised by one of the caregivers who said that it negatively affected the quality of the training.

##### Length and timing of the training

All participants reported that the training took longer than indicated in the training manual, causing childcare issues for the caregivers and difficulties for facilitators in keeping the caregivers’ attention throughout the training:

FO1: The time the programme was taking was beyond our expectation. Usually we started on time, especially the first section was long. First, when they reported their homework, we needed to patiently listen to them and give chance to all of them. This took time. Sometimes we needed to explain some issues and the questions they had. These things took a long time and as a result the training was becoming very exhausting and long … as it becomes long it is also difficult to get their [the caregivers’] attention.

The timing of the training (afternoon) was also identified as a barrier to attending the programme by some of the caregivers and programme facilitators.

##### Problems in relation to training materials

Programme facilitators reported that some aspects of the training materials, particularly the role-play demonstrations, were difficult to deliver due to their complexity and facilitator’s lack of familiarity with role-play demonstration as a teaching modality. They also indicated that the time they had reserved to practise the role-play together was insufficient, making them feel somewhat unprepared.

##### Practical challenges relating to the home visits

One of the observers who conducted home visits indicated that locating some of the caregivers’ houses was a challenge due to lack of street signage and house numbers.

#### Other challenges

##### Group dynamics: differences between participants

Programme facilitators and observers talked about the variation in caregivers’ level of understanding and education as one of the challenges of delivering the programme:

FO1: The other challenge was that the team was a mixed group. It means, some of them are more educated and have better understanding. For others, some of the issues may not be easily understandable and clear … For example, when they were paired to do a role play, one of them acting as a parent and the other as a child, they were not able to understand the instruction given to them which might be related to their level of understanding. This might indicate that they might need more support than expected …

##### Difficulty with understanding the importance of playing with a child and praising a child

The lead programme facilitator indicated that some of the CST strategies were difficult to understand for caregivers, especially the importance of play between caregiver and child (as opposed to play with siblings) and the importance of using praise as positive reinforcement:

FO1: The other is, in case of one or two families, they say that they gave the child a ball to go out and play. We explain to them that this is not the aim; they as a parent should play with him/her. Therefore, I think if it is this much difficult to explain the need for the parent to play with their child in urban areas where there is better awareness, I guess it would be more difficult in rural areas …There is nothing that contradicts with the culture or offends people. However, there is an issue of not understanding the importance of playing with their child … when they sit down with their child and play with toys, their neighbours may make fun of them and may say ‘are you going back to be a child again?’ In fact, none of the parents said this has happened to them but it can be anticipated. A parent may not be comfortable when others see him/her playing with toys with a child. Things like these might make them reluctant …

When the caregivers were reporting their homework to programme facilitators, the lead facilitator noted that, unless caregivers were specifically prompted, none mentioned praising their child. One mother expressed her concern that if she only praised her child without being tough on him, he will not be prepared for life as not everybody will treat him like her.

##### Difficulty with changing ways of disciplining a child

Although some caregivers indicated that the programme changed how they discipline their child, comments from other informants highlighted the difficulty of changing attitudes and practice within a short timeframe:

FO2: There was no content that offended them [caregivers]. However, there was a shift from the traditional caregiving or in terms of approach … the way they [traditionally] do things is if a child is refusing [to do something] or if he is disturbing, they yell at him or punish him. That part [practice] is still there with them. However, in our teaching when we teach them that punishment doesn’t work and it’s not that helpful … that may be a new change for them … I think ultimately over time they will understand it.

##### Expectation

Some caregivers indicated that not all their expectations of what they hoped the training could achieve had been met:

C9: Because I was very eager, I expected more, more things than this … eh … I expected more than this … what was in my mind is as a mother you would be eager … eh … if it is done more than this, if they are made to be more independent … eh … if the training is not finished. That’s it. I worry about her future … eh … I worry about that.

Many caregivers also expressed their concern regarding what is going to happen now that the training has finished and hoped that the support will somehow continue in the future:

C8: I am telling you the truth. I was very happy while I was taking the lessons. I felt like my life was changing and I felt like I was leading another life. Last time when we were told that only one session is remaining, honestly something inside of me dropped. I was startled and very sad. I cried when I was alone … I like what has started. It is very good. I wish it didn’t end.

C9: … what is going to happen next? We, parents came together and have raised this concern to Dr x [programme facilitator] …

### Suggestions for improvement

Finally, caregivers, programme facilitators and observers offered a variety of suggestions as to how the programme could be improved, which are summarised in Table 4. In addition, some caregivers highlighted ongoing unmet needs that are not addressed by the programme, such as financial difficulties or lack of appropriate education for their child.

**Table 4.** Suggestions from caregivers, programme facilitators and observers on how to improve the CST.

Participants	Suggestions
Caregivers	Include topics on how to toilet-train their child, how to teach their child to eat independently and how to protect their child from abuse including rape; Have more sessions
Programme facilitators	Give more support (possibly during home visits) to caregivers with lower level of understanding and education; After the training has come to an end, continue meeting them monthly or three-monthly (depending on what is feasible) to see their progress; Role-play demonstration activities need simplification; Facilitators to practise the demonstration activity well before delivering it to caregivers
Observers	Include tips on toilet training and how to look after hyperactive children; Change the training time from afternoon to morning (when some of the children are going to school, allowing caregivers to attend without having to arrange childcare)

CST: Caregiver Skills Training.

## Discussion

This study aimed to explore the perspectives and experiences of Ethiopian caregivers, professionals and other stakeholders on the WHO CST programme to inform its adaptation and implementation in Ethiopia. Findings from the consultation and review process illustrated the importance of collaborating and engaging with local stakeholders and experts, to ensure buy-in of the local community and to ensure that the programme fits the culture and context (Hoekstra, Girma, Tekola, & Yenus, 2018). Specifically, the consultation and review were instrumental in highlighting caregivers’ needs and expectations (such as the need for greater emphasis on psycho-education) and ensuring that the programme content and delivery strategy would be acceptable and sustainable in the Ethiopian setting. In addition, the consultation findings were fed back to WHO and contributed to inform the revision of CST materials, which were subsequently made available for international field testing worldwide as version 2.0.

Overall, caregivers and programme facilitators who took part in the pre-pilot of the adapted CST reported that the adapted CST was acceptable and relevant to the Ethiopian context, corroborated by the excellent enrolment and retention rates throughout the programme. Informants expressed how the programme improved care-givers’ knowledge and skills and was effective at helping them to manage their child’s difficult behaviour and their own stress and reduced social isolation. Many caregivers indicated that the training has changed their perception regarding their child’s development and the difference that they as caregivers can make in supporting their child’s development. In addition, caregivers reported that the programme gave them an opportunity to share their experiences of raising a child with DD with other caregivers and learn from each other’s experiences.

These descriptions of perceived benefits are in line with the existing literature on parenting programmes which noted the benefits of parenting programmes in understanding and managing challenging behaviour in children (Furlong et al., 2012), improving parents’ knowledge and skills (Furlong & McGilloway, 2011; Kane, Wood, & Barlow, 2007; Lindsay, Strand, & Davis, 2011), managing parents’ stress (Stewart-Brown et al., 2004) and reducing social isolation (Kane et al., 2007). Several studies (Gross, Julion, & Fogg, 2001; Kane et al., 2007; Mytton, Ingram, Mann, & Thomas, 2014) also indicated that exchanging ideas with others and feeling it was safe to talk were important for parenting programme participants.

The design of our qualitative study precludes making claims of effectiveness; rather, these comprehensive descriptions from caregivers about the perceived benefits of the programme can be interpreted as evidence of acceptability of the programme (Sekhon, Cartwright, & Francis, 2017). Previous studies in Ethiopia (Tekola et al., 2016) and other low-income countries (Scior et al., 2015) have highlighted the common belief that children with DD are incapable of learning. The pre-pilot results give a preliminary indication that the CST programme may be able to challenge this belief and instil a greater sense of hope in families.

The findings of this study in relation to challenges and barriers to participating in, delivering and implementing the CST programme are in large part consistent with the extant parenting programme literature. A systematic review of qualitative studies regarding facilitators and barriers to engagement in parenting programmes highlighted barriers like the issues raised in our study, including group dynamics, competing demands on parents’ time and resources including child care for other children, and lack of family support to attend programme (Mytton et al., 2014). Similarly, in a review of qualitative evidence on factors blocking or facilitating access and engagement of parents with parenting programmes, Koerting et al. (2013) indicated that practical issues such as difficulties with transport and finding childcare were commonly reported.

However, some of the challenges and barriers reported in this study are seldom discussed in the existing parenting programme literature. This is particularly the case in relation to caregivers being unfamiliar with the idea that playing with their child and praising their child can help their child to learn, and caregivers’ reluctance to completely abandon their ways of disciplining their child (such as yelling and spanking) in favour of more positive disciplining techniques. These findings suggest the importance of considering socio-cultural contexts in implementing and delivering parenting programmes.

Caregivers and programme facilitators/observers had several suggestions for improvements to the programme. Caregivers’ suggestions focused on including additional topics to the programme such as toilet training, independent eating and protecting children with DD from abuse. Toilet training is intentionally excluded from the WHO CST as it requires a specific focus which is beyond the scope of the programme. This study suggests that there is a demand for further resources specifically targeting toilet training. Programme facilitators/observers’ suggestions generally focused on mitigating the challenges and barriers in relation to participating in, delivering and implementing the programme. One of their suggestions, for example, was providing ongoing support to caregivers after the completion of the programme. This, to some extent, would help to alleviate caregivers’ concerns regarding what is going to happen after programme completion.

Some of the findings of this study may inform the adaptation and implementation of evidence-based interventions in other low-resource settings. These include first, the importance of involving relevant local stakeholders (including caregivers and local experts) in the adaptation process. The involvement of local stakeholders is critical in order to understand the local needs and context and to identify socio-cultural barriers that may affect the implementation process of the programme, and how to overcome these barriers (Dababnah, Habayeb, Bear, & Hussein, 2018; Hoekstra et al., 2018). For example, this study highlighted caregivers’ reluctance in using praise as positive reinforcement, and suggested not all caregivers may be aware of the importance of play between them and their child in supporting their child’s development.

Second, delivering parenting programmes in low-literate, low awareness and high stigma settings may present another layer of challenges which should be addressed such as parental guilt, stigma, unhelpful beliefs (e.g. that a child with DD is unable to learn), expectation of a cure and physical punishment. In such settings, parenting programmes should also include increased focus on psycho-education and strategies for teaching low-literate caregivers (e.g. greatly simplifying participant booklets and discussing concepts orally rather than relying on written text). In addition, in settings where caregivers experience severe social exclusion and where there is low awareness, childcare at home may not be feasible. Childcare may need to be arranged at the programme site and this has clear resource implications for running the programme.

Finally, in a context where no other services are available caregivers may have great expectations and worry about what is going to happen next after the completion of the programme. In our study, participating caregivers expressed their need for ongoing support and to continue to meet and interact with each other even after the completion of the programme. In response, we arranged a venue for them to meet up monthly. The importance of setting up parent support groups for parents of children with autism in low-income settings was also highlighted in a study in Bangladesh (Blake et al., 2017).

## Limitations

Although this study explored the adaptability of the WHO CST in both urban and rural Ethiopian settings, the adapted CST was pre-piloted only in an urban clinical setting facilitated by specialists. We recognise that different benefits, barriers and recommendations in relation to participating in, delivering and implementing the programme may be relevant in less-resourced rural settings, with non-specialists and with caregivers who have never received any kind of service before. In addition, although social desirability bias in qualitative interviews was minimised by using an independent qualitative researcher not involved in CST delivery and encouraging participants to give their honest opinion and share any critical feedback about the programme, we cannot completely rule out socially desirable answers. It is also possible that in a low-resource setting, where no other services are available, participants may be more likely to evaluate any programme positively.

## Conclusion

The adaptation of the WHO CST to the Ethiopian culture and context involved a comprehensive process which included multiple consultations and interviews with Ethiopian caregivers, professionals and other stakeholders, detailed review of documents, continued communication with the WHO CST team and translation of materials to the local language. The pre-pilot study findings suggest that the WHO CST programme addresses a local need and, with careful adaptations, is feasible and acceptable to be implemented in urban Ethiopia. The findings described in this article can inform the adaptation and implementation of interventions in other low-resource settings.

## Supplemental Material

AUT848532_Lay_Abstract – Supplemental material for Adapting and pre-testing the World Health Organization’s Caregiver Skills Training programme for autism and other developmental disorders in a very low-resource setting: Findings from Ethiopia Supplemental material, AUT848532_Lay_Abstract for Adapting and pre-testing the World Health Organization’s Caregiver Skills Training programme for autism and other developmental disorders in a very low-resource setting: Findings from Ethiopia by Bethlehem Tekola, Fikirte Girma, Mersha Kinfe, Rehana Abdurahman, Markos Tesfaye, Zemi Yenus, Erica Salomone, Laura Pacione, Abebaw Fekadu, Chiara Servili, Charlotte Hanlon and Rosa A Hoekstra in Autism

AUT848532_Supplemental_material – Supplemental material for Adapting and pre-testing the World Health Organization’s Caregiver Skills Training programme for autism and other developmental disorders in a very low-resource setting: Findings from Ethiopia Supplemental material, AUT848532_Supplemental_material for Adapting and pre-testing the World Health Organization’s Caregiver Skills Training programme for autism and other developmental disorders in a very low-resource setting: Findings from Ethiopia by Bethlehem Tekola, Fikirte Girma, Mersha Kinfe, Rehana Abdurahman, Markos Tesfaye, Zemi Yenus, Erica Salomone, Laura Pacione, Abebaw Fekadu, Chiara Servili, Charlotte Hanlon and Rosa A Hoekstra in Autism
