# Supplementary material for: Adapting and pre-testing the World Health Organization’s Caregiver Skills Training programme for autism and other developmental disorders in a very low-resource setting: Findings from Ethiopia
Source: Autism. 2019 May 16;24(1):51–63. doi: 10.1177/1362361319848532 (PMC6927066; doi:10.1177/1362361319848532)
Supplement: AUT848532_Lay_Abstract – Supplemental material for Adapting and pre-testing the World Health Organization’s Caregiver Skills Training programme for autism and other developmental disorders in a very low-resource setting: Findings from Ethiopia [file AUT848532_Lay_Abstract.pdf]

autism

DOI: 10.1177/1362361319848532

Adapting and pre-testing the World Health Organization's Caregiver Skills Training programme for autism and other developmental disorders in a very low-resource setting: Findings from Ethiopia

Authors: [paste here]

In low-income countries most children with autism or other developmental disorders (DD) remain undiagnosed and receive little or no formal help. To address this need the World Health Organization (WHO) has developed a Caregiver Skills Training (CST) programme that teaches caregivers strategies to help them support their child. Before the programme can be rolled out globally it is important to test whether it suits families' needs and is feasible to implement in very low-resource settings like Ethiopia. We first conducted an extensive consultation and review, asking Ethiopian caregivers, professionals and other stakeholders their views on the draft CST materials. Based on this feedback the materials were adapted to fit the Ethiopian context and then translated. We subsequently pretested the programme in Addis Ababa and interviewed all participants about their experiences. All families who enrolled completed the programme, suggesting high acceptability. Participating caregivers perceived the CST to be helpful in furthering their understanding of their child's needs and how to support their child's skills development. They also valued sharing their experiences with other caregivers and indicated the programme helped them manage their stress. Challenges were reported too: many parents found it hard to find childcare; some found it difficult to find time to practise what was learned at home. Programme facilitators reported that some CST strategies are unfamiliar and difficult to understand for caregivers. In conclusion, our study suggests that the WHO CST programme addresses a local need and, with careful adaptation, is feasible and acceptable to be implemented in urban Ethiopia.
