# Supplementary material for: Adapting and pre-testing the World Health Organization’s Caregiver Skills Training programme for autism and other developmental disorders in a very low-resource setting: Findings from Ethiopia
Source: Autism. 2019 May 16;24(1):51–63. doi: 10.1177/1362361319848532 (PMC6927066; doi:10.1177/1362361319848532)
Supplement: AUT848532_Supplemental_material – Supplemental material for Adapting and pre-testing the World Health Organization’s Caregiver Skills Training programme for autism and other developmental disorders in a very low-resource setting: Findings from Ethiopia [file AUT848532_Supplemental_material.pdf]

Supplementary materials

Topic guide for interview with caregivers post CST pre-pilot testing

Family ID________________ Participant ID: _____________________

Family Member:

- ☐ mother ☐ father
- ☐ grandmother ☐ grandfather
- ☐ sister/stepsister ☐ brother/stepbrother
- ☐ other (specify) ___________________________

Date:____________________

Location:_________________

A. Introduction

Interviewer to say his/her name and where they are from.

We are here to learn more about your experiences taking part in skills training for caregivers of a child who develops slowly. The information you give us will help us to further adapt the training as necessary, so that it meets the needs of families in Ethiopia.

While we are talking we are recording what is said with this tape recorder so that we can remember all of the information. Everything we learn from people will be put together so that we have an idea of the views and experiences of many different families. There are no right or wrong answers.

B. Interview guide

[Start tape recording from here, start tape recording with saying the family ID number, but not the name of the caregiver]

- Ask about experiences with programme, both positive and negative.

Please tell me about your experience of the programme

What did you like about the programme?

What didn't you like about the programme?

- Explore the usefulness of the programme to the caregivers.

Tell me about how relevant the programme was to your circumstances."

How useful was the programme? In what ways was it useful?

Did the programme help you to feel more confident? In what ways?

Did the programme help you to feel less stressed? In what ways?

Did the programme help you to improve your child's skills? In what ways?

Was the programme useful in ways that you did not foresee or expect? Please tell me in what ways?

- Explore usefulness of CST components and strategies.

What part of the programme was most useful to you? Why?

Tell me about your experience of the home visits? What were the positive and the negative aspects?

How did you find the videoing?

Tell me about your experience of group sessions? What were the positive and the negative aspects?

Which strategy that you learned was the most useful? Why?

- Ask for suggestions to improve relevance and usefulness of CST content.

What else would you have liked the CST programme to address?

What other important messages do you think need to be conveyed to caregivers of children with developmental delays or developmental disorders?

- Explore acceptability of programme content.

How did you find the messages and stories that were used in the CST sessions? How much did those messages and stories fit with your situation?

- Ask about experience and difficulties with implementing the strategies at home and suggestions to make home works more feasible.

What made it hard to implement the intervention with your family? What helped to make it easier?

- Explore barriers with attending the programme and suggestions to improve attendance and reduce drop out.

What made it easy/hard for you to attend the training sessions? Any suggestion to make it easier to attend?

- Explore programme impact on families.

What kind of impact has the training had on you? How about on your child? How about other members of your family? How about on relationship among family members

- Explore if the programme implementation has triggered any change in the community.

How do you find getting out and about in your community? Has the programme affected that in any way? Please tell me about that. [Probe about awareness, accessing support, stigma]

- Explore adverse effect on caregivers, families and communities.

Did you or your family experience any difficulty or challenge or discomfort in relation to attending the CST programme?

Have you experienced any negativity from your community since the start of the CST programme implementation? [If so, ask for suggestions to avoid this in the future.]

- Ask for any additional suggestions and thank participants.

Is there anything else you would change about the programme?

Are there any other thoughts or comments you would like to share with us?

Topic guide for interview with CST facilitators and CST observer post CST pre-pilot testing

Participant ID #: ☐☐☐☐☐

Date:___________________

Location:________________

A. Introduction

Interviewer to say his/her name and where they are from.

We are here to learn more your experiences delivering the parent skills training programme for caregivers of children who develop slowly. We would like to hear from you which parts of the programme worked well in your opinion and which parts could be improved. We would also like to hear if you experienced any challenges in delivering the programme and would be grateful for any suggested revisions you may have.

The results from this study will inform the further adaptation of the training programme, to tailor it to the needs of the Ethiopian setting. The results from this study will also be fed back to the World Health Organization, so that it can inform the adaptation and implementation of the programme in other countries.

While we are talking we are recording what is said with this tape recorder so that we can remember all of the information. Everything we learn from our interviews with the CST facilitators and CST observer, as well as from the participating caregivers, will be put together so that we get a broad overview of the views and experiences, and this will inform the revisions we will make to the programme.

B. Background

First we would like to ask you some background information.

1. What is your gender? Male/Female

2. What age are you now? _________ years

Date of Birth (if known) [ ]

Today's date [ ]

3. What are your educational qualifications?

____

4. What is your current profession? _____________________

5. How long have you been working in your current job role?

________months __________years

6. What was your role during CST implementation?

Lead facilitator [ ]

Assistant facilitator [ ]

Observer [ ]

7. Do you practise a religion?

No religion	[ ]	Orthodox Christian	[ ]
Catholic	[ ]	Protestant	[ ]
Muslim	[ ]	Other	_________

C. Interview guide

[Start tape recording from here, start tape recording with saying the participant ID number, but not the name of the informant]

- Ask about experiences with programme, both positive and negative. Can you tell me something about your experiences with the programme?'

What were the positive aspects?

What about negative aspects? Any difficult experiences? Please tell me about these?

[Prompt about difficult experiences with engaging participants, conducting home visits, facilitating any activity, making the programme relevant to the group of caregivers, how confident/competent they felt, time burden, emotional burden, handling expectations/requests (e.g. for money) of the caregivers]

- Ask about acceptability of the programme content.

Was the content of the programme acceptable to the caregivers? Did any aspect of the programme offend participants or made them feel uncomfortable? How might we improve the acceptability of the materials?

- Ask for suggestions to make the materials more useful to caregivers and families.

How were the topics in the programme relevant to the caregivers? How were the examples in the programme relevant to the caregivers? What could make the programme more useful to all caregivers and their families?

- Prompt for additional suggestions for change in the programme materials.

Which activities would you suggest omitting in the future? Is there anything the programme that was missing that would have been helpful for the parents you worked with?

Would you suggest any other change or adaptation to the CST materials or strategies (any change to the CST facilitator guides, participant booklets)?

- Ask about perceived impact of the programme on families.

Do you think that the programme helped parents to feel more confident in their parenting? In what ways?

Do you think that the programme helped parents to feel less stressed? In what ways?

Do you think that the programme helped parents to improve their child’s skills? In what ways?

To your knowledge, did any caregiver, child, family or other in the community experience any problem because of the CST programme?

- Ask about barriers to implementation.

What has made the implementation of CST more difficult? What could have made the implementation of CST easier? [Probe – organising the sessions, finding a room, childcare availability]

Would you suggest any change or adaptation to the CST mode of delivery (any change to the delivery in a group setting, home visits, delivery by two facilitators (one lead, one assistant)?

- Ask about suggestions to improve strategies for training and supervision.

Did you feel well prepared to deliver the CST? Would you suggest any change in future training of CST facilitators? (or, if the informant is the CST observer rather than the facilitator: After observing the CST being delivered, would you feel well prepared to deliver a similar training yourself? Is there any additional training you would benefit from before taking on this role?)

How supported do you feel? Would you suggest any change in mechanisms for supervision?

- Ask about the usefulness of home visits.

Did you conduct any of the home visits? [if answer is 'yes', follow up with the following questions: How useful were the home visits? How useful was the video-recording during home visits? Were there any issues with video-recording? Please explain.]

- Ask for any additional suggestions and thank participants.

Is there anything else you would change about the programme?

Are there any other thoughts or comments you would like to share with us?

Supplementary material:

Theme 1: Acceptability and relevance of the programme

Full quotes from all participants relating to theme 1 are presented in the matrix below. Quotes included in the main manuscript text are highlighted in yellow.

Participants	Acceptability and relevance of the programme
C1	…the training did not have any shortcomings. If I am not repeating myself, it was very effective. It is good, and I am happy about it. But still there are things that need to be improved. I am still thinking of one thing, which is, it would be better if five or ten parents could attend the training together for certain days. You come from home and attend a class, this is very difficult. Thus, it would be nice if they could attend for some time. In addition to this, now we just come here, take the lesson and get back home…of course, this might help us to give direction for our children, but these children are growing up and the thing is…of course I am talking out of the topic, but it would have been nice if something could be done for our children. For example, it might be a school or a place for them to stay and to develop their mind…
C2	…I attended the training with many other parents happily ...eh... it was not boring. It was not stressful. They [the CST team] were supporting us with lots of things by being on our side. I don't have any complaints …we had a very good time. Nine weeks felt like a week…
C3	The training was very useful for me. I didn't know how to handle my child before this training…
C4	I have attended all [sessions]. It was good for me… [during the first home visit] because my house is very small, I was not comfortable for them [CST assistant facilitators] to come but it is good…. everything is good….
C5	The training enabled me to know things which I didn't know before and it enabled me to raise my awareness. I got many lessons from the training; it was excellent. I have great respect for the training…
C6	…the training was advantageous in that it gave us knowledge and we are working with it…it would be good if it could be continued. It was very useful for us…
C7	…Do you know what I consider as difficult? The case [condition] itself. Otherwise, the things we have learnt, we have learnt them in a simple way to improve the children, the case itself is difficult. The teaching is good…
C8	…first of all, I want to say thank you. I have learnt a lot from the training. All the things that we learnt were essential for me. I want to tell you that it has changed my life. I am happy with the training, I have accepted it. In general, it is hard for me to express, from the beginning to the end, it has changed my life…there is nothing that I believe should be changed [in the participant booklet] but I say it should be added. I would be happy if more things would be added… I didn't expect that they [the CST team] will know what we were feeling deep down and they would present it to us in the lessons. I didn't know that they knew [what we were feeling inside] … I came here just to check it out… when I took one class, I saw a lot of the things that were in my life. All the problems I had at home were written down in this book [participant booklet] …<br>… … I was happy to come [to the training] leaving everything behind. There is nothing more important for me than this issue. Honestly, for me there is nothing more important than my child. If there is something which helps him to improve, I will leave behind everything [if necessary].
C9	There is no part which should be discarded. This is my personal opinion. Before this training, I took lesson from my child and after I started the training, the training increased what I have internally. I had awareness before because I have been living with my child, but the training developed my awareness…
FO1	I don't believe that the programme has negative sides. As per my experience, eh generally it does not have something that contradicts with the culture. I have not found anything that contradicts with the culture or something which in one way or another hurts their feelings or something which disappoints them. If there were such things, I would have taken them as negative aspects [of the programme] …one other thing I have observed is that all parents, as far as I know, have attended all

	the sessions… When one parent could not come, he sent his wife to attend the training…this shows how they have taken the programme seriously and believe that it is very useful for them.
FO2	Most of the contents that they have learned are appropriate and useful. They have gained useful lessons that they can apply for the betterment of their children…. According to the feedback we got from the parents, the contents included [in the training] are very relevant and appropriate. As far as I am concerned, there was no content which is not relevant, or which made me dissatisfied….
FO3	The good aspect about the training is it fulfilled the parents' need. Starting from the beginning, the lead facilitator has clearly explained to parents what they are going to get out of the training. It teaches them what they need to do for their child. It was very useful, and the parents were very happy from the beginning to the end…
FO4	…the mothers were fully ready for the training…they believed that from the training they will gain additional knowledge and skills that would enable them to look after their children better, this is the first and major thing for me…all the issues included in the training were very good and useful. There is nothing missing. It included everything important, so I would say should continue as it is.
FO5	when you look at it as a start, this parent skills training was very good. I think in my opinion it was good…eh the families were very happy. They were attending the training happily. When they gave comments and suggestions at the end of the programme, they were happy with it. The did not say anything negative except the time it took considering they were coming from far places…they found all the sessions useful. As they told us, everything was very good and appropriate for them….it is good if the contents remain as they are eh there is no section that they did not like. Most sections were appropriate for them and when they practice at home, they did not find it difficult, so it is good if it continues as it is.

Supplementary material:

Theme 2: Perceived benefits of the programme

Sub-themes 1: Improved knowledge and skills

2: Positive effects on psychological wellbeing

3: Changed perception

4: Sharing experiences with other caregivers

Full quotes from all participants relating to theme 2 are presented in the matrix below. Quotes included in the main manuscript text are highlighted in yellow.

	**Perceived benefits of the programme**			
	**Improved knowledge and skills**	**Positive effects on psychological wellbeing**	**Changed perception**	**Sharing experiences with other caregivers**
C1	…what I liked the most about the training is, the experience…I have gained lots of experience because of the training…<br><br>…I have obtained many experience… my child has improved compared to the previous times, as I am taking care of him properly…That's why I generally told you that the training has helped me a lot. It helped me to improve many things…so many things…			
C2	From the training the good experience we took is, until now we did not have the awareness that these kinds of children can improve. Now Professor x taught us lots of things in a very good way… We have learned that our children can improve and that they can change… I usually do things for my son thinking that he can't do it by himself. When I change him, when I feed him, I pressurise him but now because of the training I took, I think my child can do things and I am by his side to support him when he can't do things ... Eh ... We have learned that we need to ask ourselves who we are, to give time to ourselves and take care of ourselves and that it is when we take care of ourselves that the children will have a chance to live well ...Eh... We have learned that if we [the parents] are not well, the children will also not be well. We did not know all these before…	…I don't know how to express it to you, there is a lot of burden. Even if we are in pain, we tolerate it. There are lots of things ... eh... I am raising my son without a father ... eh... I have a heart condition but now thanks to God it is getting better. I am getting better ... eh... When I get stressed, it relapses but now I am getting a little better. I am taking the medication. Thanks to God. When I meet with lots of parents eh… I have stopped being stressed and I say there are lots of changes…	… [After we took part in the training] we have changed a lot…eh…I was hopeless…eh I used to think that my child can't mix with other people. Now my child has come out and I also came out, many people have seen us. We met with many parents. We shared with each other our pains and many other things and I believe we are going to bring about many changes… I don't know how to explain it to you. I just can't. God has something better...eh... We are not going to stay like this. Our children may change after our effort. After an effort a person reaches at a certain outcome. We hope we reach at a certain outcome…eh…I am doing everything I can thinking that I can bring change for him [my child] …	
C3	The training was useful…It helped me how to handle and care for my child…. Doctor x taught us in a very good way. He told us how to handle our children, how to care for them… I have learnt many things. It is difficult for me to say this and that. It is very good. It has changed me a lot…	The training was very useful for me. I didn't know how to handle my child before this training. I always felt stressed. He didn't know how to put on his clothes quickly but [each morning] I ordered him to put on his		

		clothes quickly because if it is 8:30am I may be late for my work… even when we walked on the street, I usually told him to walk fast… I used to get stressed and made him stressed. Now this lesson has helped me a lot. I now know lots of things. Now I calm myself down and help him to get dressed. It has helped me a lot.		
C4	It is good for my child. They told me to keep his personal hygiene and to play with him. I am telling him what they told me. …previously my son did not dress himself…now he pushes one of his arms through one sleeve and I help him with the other sleeve. He puts on his shoes by himself and I tell him to put them on the right feet…it [the training] is very useful.	…Now I am mixing up with people. Previously I did not mix up with people. I did not mix up with anybody. I used to lock my door and stay at home and only open the door when my children come home from school. I am good now because they [CST facilitators] are encouraging me when they come for the home visit…		
C5	The training enabled me to know things which I didn't know before and it enabled me to raise my awareness. I got many lessons from the training; it was excellent. I have great respect for the training…My child has a speaking problem. He is not calm and couldn't sit at one place. This training helped me how to teach my son to start speaking. Before this training, we [my husband and I] used force to make him do things. But now we received lessons about this and since I understand now how to help him say words, thank God now my child starts to say words. He is asking what he wants…when he wants injera [Ethiopian flatbread] he says, 'give me injera' and when he wants water, he says 'give me water'. This is a big change and I am very happy…after the training we received lessons about the importance of appreciation. Based on this, we started appreciating our child. Before the training, I didn't know that a child becomes happy when we appreciate him… My families also accepted the lesson that I received, and we are working together to teach my child…He couldn't wash his hand and face previously but now we give him water and he washes by himself…The other thing is he is also brushing his teeth…I am not saying he is able to brush his teeth properly, but he tries. This is not a simple thing…We are teaching him to put on his trousers and socks by himself. He does not do these tasks properly, but he is trying…The teaching helped us to know what to do and how to do it… when I ask him to do something, he does it for me. I say the name of the object and ask him to bring that object for me then he brings that object for me. For example, when I ask him to bring water, he brings water. When I ask him to bring my shoes, he brings my shoes. This is not a simple thing…Before I took part in the training, as I told you, I used to yell at him [my child] and as a result he feared me, and he only approached his sisters. He was also scared of his dad as he shouted at him even		The training was good. The first thing is it helped me to live with confidence. By the way, I left my work because of my son. I am now more convinced that my child can change. I have more confidence now. I have changed a lot…	…we were able to share experiences with other parents. There were some strong mothers and there were those who did not understand [what was being said]. I needed to share with them what I know so that they can also know. Through time they will be fine. If they didn't come out of their house they wouldn't get this [opportunity]…

	more than I did…the training was useful for me because my child is now closer to me than before…a child needs love…			
C6	…the training benefited me [in terms of], first, when I ordered her [to do something], I didn't say it firmly 'you need to do this', there was a tendency to hesitate. Now, however, I learnt that I need to tell her firmly and encourage her by giving her rewards by saying I will do something for her. I understood the benefits of this after I took the training. Secondly, the need for me to encourage her. Earlier, I used to wonder how I would get through the future with her condition, for how long I would be able to handle it. I used to say, 'as she gets older, I wouldn't be able to handle it'. I used to only use force, when I yell, I get upset and made the neighbours feel uncomfortable. But after I took this training, it very much benefited me in terms of how to calm her down and manage her…she always wanted to watch the channel that she wants. Now, we don't do it like this, we also want to watch TV. Before, if I say that to her, she shouts a lot, she just wanted to do what she wants. But now I give her a warning, we give her the message that she is only going to watch for a certain time. I have now understood how to do this. Earlier, I did not know how to handle the situation… the training was advantageous in that it gave us knowledge and we are working with it.	Earlier when there were problems, I did not have patience. I mean, I did not have the calmness when she shouted and made noise like that, I used to get upset too. It [the training] has helped me in terms of how to first keep myself calm to be able to calm her down……[earlier] when my husband hit her and shouted at her I used to feel something inside. I used to tell him that she did not want to do this, she is not like this on purpose and that we need to understand her. I have been through a lot for her…when my husband spoke angrily to her, I used to feel sick and anxious, I used to get very upset. when he started understanding the training… he says this is how we should have treated this child. He understood now, he understood that she needs care, now, we are showing her more love to the best of our ability…		
C7	The training is very good, and we think it is very useful for our children. Earlier, I don't tell my child anything and I preferred to be silent. But after I received lessons from this training, when she asks me questions, I explain to her, she will say 'do you know the name of this?' I reply based on what I know. Earlier, she couldn't put on her cloth but now she is trying even though she didn't put it on correctly… now I am giving her simple clothes and she is trying to put her clothes on. When she needs something, she will ask me, and I will respond based on the lesson that I got from the training and the advice that I got from Doctor XX…	I take things lightly now. That means I don't interfere in everything she does. Previously, I said to her 'stop you can't do that'. Now I see it as something she can do, I show her and if the thing she does is wrong and if it is not something which hurts her, even water or something that she eats, if it is something that does not hurt her, I let her do it her way and take it easy. Previously, I would shout at her.		…I received many lessons from the parents. We have learned from each other's experiences. It is very good. The lessons I got from the parents are good.
C8	…I have learnt a lot from the training. All the things that we learnt were essential for me. I want to tell you that it has changed my life. I am happy with the training, I have accepted it. In general, it is hard for me to express, from the beginning to the end, it has changed my life…For example, when I discovered about my child's situation. I was someone who had no clue. I didn't know anything about the topic. I didn't know anything about autism and I was so scared. I didn't have a good thing.	…any mother let alone a mother with this kind of child, you worry about your child. About tomorrow and today and things like that. His [my child's] problem was even big. After I took the training, I have changed. I am telling you the truth. I got self-confidence. I	…I have been through a lot. I have suffered a lot. After I came here lot of things changed be it confidence, I am no longer scared, I am not worried about tomorrow. I am telling you the truth. I have learnt that I can take care of my health and take care of him [my child]. I	…It's a big deal to be able to share with people about your experience. When you can share the things that you didn't even share with your family you become very happy. It has made me happy. I have got many things from the programme. I don't regret it. I

	I didn't have a good sleep I didn't have good life. I was really startled and didn't know what I was going to do…I have learnt a lot. How to treat him [my child]. And even how I should lead my life after this, they have thought me everything. Along with Allah based on what I have learnt I am ready to implement everything…because I didn't understand my child's behaviour, I used to think that he was spoilt, or I was a weak mum. When I get angry with his behaviour, I used to think that it is because I am a bad mum. I couldn't handle it. I used to think that was my problem. I used to hit him a lot so that he keeps quiet. [Because of] his behaviour, along with the regret and wondering why he became like this, I used to get angry a lot. Honestly, now I get sad because I used to hit him. Every day I get sad because I used to hit him. What can you do? You can never take it back but after I took the training, I have not raised my hands to him. I always regret what I did. That was because I didn't have the knowledge. After the training, I take everything seriously be it his food, or when he does something wrong now I understand that he doesn't do it intentionally…	realised I can do a lot for him…I used to think that there was something wrong with me. All of that was removed. Honestly, things have become a lot easier. Things are easier. I am happy all the time. I realised things can be better. I wish I received the training earlier…	have learnt that I can do lots of things and I am very happy, it's honestly hard for me to express…To be honest with you it is me who has learnt more than my child. Now, I very much have hope. I feel like I can teach him from the beginning; I feel like I have the knowledge…	am very happy…I used to say that something needs to be added [to the training manual]. But now do you know why I no longer say that? I now realise that every child is different. Previously, at the beginning by focusing only on what concerns my child, when parents in our group talk to each other we used to say that this thing does not concern my child. We used to say this among ourselves. But when you listen to what other parents experienced you realise that everything is there [in the training manual] …at the beginning I used to think that only the teaching is beneficial to me, but I realise that the parents experienced and talked about different things [which I did not know about]. They have experienced what you have experienced, or they are talking about an experience which you have not yet gone through. So, you learn so many things. It is very good.
C9	The first thing is the training helped me to be calm…I now understand how to help my child. Previously, both of us [me and my child] used to shout at the same time but after this training I understood that I need to give time to her and to listen to her. The other thing is I have also learned to increase the number of words I use with her. We use words when she puts on her clothes and I will ask her what she wants. Generally, the training was useful, and it was very good…Before this training, I learned from my child and after I started the training, the training increased what I have internally. I had awareness before because I have been living with my child, but the training developed my awareness. The training gave me strength… I worked alone in the previous times but now we are working together [me and my child]. It may be washing dishes, clothes etc.…	The first thing is the training helped me to be calm. I am free from stress…	…especially for me it has given me strength…eh…it made me to think that I have hope… The part I liked the most was: about my life...eh…accepting it [my child's condition] …eh...calming myself…eh…that is it…	We [the parents] share what we know among ourselves. What I know may be something the other parent doesn't know and what I don't know may be something the other parent knows… the life I have experienced may not be the life they experienced …eh… I have learnt a lot because of my child…eh…eh...do you understand eh we have experienced so many difficult things…
FO1	…I realised that the parents or caregivers I trained lacked very basic information. I recognised that the information included in the training was very useful, because things that seem basic and simple but which the parents didn't understand were included. In addition to the basics, I think there were things that can be helpful to the parents or caregivers with their day to day life, to be able to help their children achieve useful skills and knowledge…		…apart from acquiring skills, it [the training] also helped them to correct the negative attitude they get from different sources. To give you an example, every one's children can grow, every child can develop. This is very useful information because, for most of them, traditionally or medically when they are told that the diagnosis is developmental disorder, [….] they don't have an expectation that their child can develop further. They have an	…The other is, I think they [caregivers] benefited from coming here and discuss about their problems informally among themselves during tea breaks and when they arrive early. That helped them. When discussing with each other, some parents have their own experience which they share. These are experiences that they have tried by themselves which

			attitude that it is impossible for the children to improve and make changes. Therefore, it was very useful and positive in this respect... As I said before, every child can develop. I think this is a big lesson. Instead of just accepting and sitting doing nothing about the situation the child is in, attitudinal change made the parents take a step, thinking that the child can learn and be actively doing something and engage in things that their child can learn. Some parents were very happy. They say that their child was used to not doing this and that stuff, but now s/he is doing it. They say that they used to not let their child do the household chores, but now when they try them, they can do it. There were very positive feedbacks…	are not part of this program which can be helpful to the others….
FO2	…the training has lots of positive sides. It provided the parents of children with developmental disorders appropriate teachings. It provided teaching on how to care for and help their children. It has taught lots of skills to the parents. Most of the contents of what they have learnt is appropriate for them and their children…In addition, ah….it has thought the parents how to control their emotions and become better parents and better caregivers. And from the first session when you see their approach with their child…. ah…. plus, when we see their own situation and when we compare it with the last [session] there is a very big difference in terms of treatment of their child, taking care of themselves, controlling their emotions. Plus, they tell us in their own words about their experience and the things that they have gained are big things for them and it has changed them …. ah…. they tell us that their children have also learnt a lot and have shown lots of change …. ah…… the training has even gone more than expected. It was perceived that change will come after a long time. But within these sessions …. ah…. we saw changes over short period of time …ah…. from what I have understood these are all good things.	…there were lots and lots of changes on the caregivers as well. Starting from their appearance, their emotions, in terms of every way, starting with their mood lots of changes were observed. Maybe what has supported them is the fact that they have shared with each other. The feeling that they are not alone and the feeling that there are people with me and there is a concerned body has such kind of message… What surprised me the most was when we were rounding up to the last session, without even the need for that session they have already started doing proper things on their own and were returning to their normal self. This indicates the extent of their progress and might serve as a confirmation.		…there were lots and lots of changes on the caregivers as well. Starting from their appearance, their emotions, in terms of every way, starting with their mood lots of changes were observed. May be what has supported them is the fact that they have shared with each other. The feeling that they are not alone and the feeling that there are people with me and there is a concerned body has such kind of message…
FO3				…The thing they share and discuss in the group session was very good. This is because firstly it gave them a chance to share with each other what they have learned. The assistant facilitators observed the discussion by going from one group to the other. Thus, it was good.

FO4	…When we were doing the home visit, mothers were hopeless and didn't know what to do, they went to the hospital that they know… but didn't know in detail, they were not sure whether it is autism or another problem that their child has. We talked with the mothers, step by step before they came to the training. First, we tried to add a little knowledge on their attitude and then they came to the training. After having two or three sessions of the training, they started to see that they were wrong, they knew the reason why they were wrong. They think it was because they don't have the knowledge or don't have enough knowledge about it. When they get the knowledge, they tried to improve each step they have taken previously…		I have worked on many mother related things…many mothers are focused on the problems that they face; they don't think that they can improve their children's condition. What I have seen here is that, even though they still get some benefit, instead of the small benefit they get they try to gain knowledge and change their children. I have been here for all the nine weeks, in this small period the results are the changes in the attitude of the caregivers. They started to think of changing their environment for the comfort of their children… at the end, what I have observed is courage. Courage which says even if you [CST facilitators] are not going to be with us all the time I will walk through life with my child.	…at the end [of the programme], that is the 8th and 9th weeks, mothers I have mentioned earlier [those who were hesitant to speak up] were showing some sort of enthusiasm and increased participation when other parents share their experiences with them…There was this mother that I remember she tried her best and explained to us that her child is not doing what she expects him to do, she said that this training is not working well for her. The other mothers shared their experiences and she went home and tried that as well and slowly observed the changes she aspired and came back with a different story…
FO5	… from what they told us, previously there were parents who had no awareness about the topics discussed in the training. For example, about how they can communicate with their children, how children with developmental delay communicate, for example it could be by crying or by showing different behaviours. They used to punish them when they do that...however after the training if that thing happened, they ask 'what happened earlier that made him to be like that'…the other is they got awareness that they have to work on each of their skills every day for two to five minutes. After practically doing that they observed a progress…		What I observed is that: accepting their child's condition. I mean I noticed this after the training, around the end of the training. Initially, they didn't accept their child's condition. There were families who didn't have any idea about what to do…they [the caregivers] are confident that their children will improve, based on what they have learnt, they are confident that their child will show progress, although progress may be slow…	

Supplementary material:

Theme 3: Challenges and barriers (part 1)

Sub-themes:

- Participant challenges
  - Practical
    - Childcare
    - Transport problem
    - Difficulty with implementing what they have learnt at home with their child
  - Social and cultural barriers
    - Lack of support from family to attend the training
- Programme delivery challenges (presented in another table)

Full quotes from all participants relating to theme 3 – participant challanges are presented in the matrix below. Quotes included in the main manuscript text are highlighted in yellow.

**Challenges and barriers**				
**Participant challenges**				
**Practical issues**				**Social and cultural barriers**
	**Childcare**	**Transport problem**	**Difficulty with implementing what they have learnt at home with their child**	**Lack of support from family to attend the training**
C1	…it's not possible to attend the training keeping my child here [in the training room] because I will not attend to the training properly…I remember one parent, she usually brought her child here but I don't think she paid attention to the training properly because, I am sure the child disturbed her as he has a problem and you can't blame him for that…I even saw a child who came in the class room and hits people while we were attending the training…because they couldn't keep him there [in the play room next door]. So, it's better if such things don't happen [in the future] …it would be nice if the training could be given to us in a short form within three or four days in the presence of both parents and their children. This is my personal suggestion.	transport is one of the problems…after we stayed here up to 4pm I found it difficult to get transport to go home. I arrived home late at night. If my [spouse] was not there to look after my child who would look after him? That is very difficult.	I have not implemented all of what I have learnt at home. That is due to lack of time. I have three children.	
C2				
C3	I don't trust to leave my child with neighbours. If I want to go to the countryside [leaving him behind] I worry about him. They won't treat him like I do. He can't express what he wants. He may get hurt. So, if I leave my child with other people, I worry a lot. I don't trust them. He is my only child. I don't have any other child…		I have work burden. I wake up in the morning and take him [my child] to school. Then at 3pm I go to his school straight from my workplace… to bring him home from school. When I arrive at home, I need to do all the household chores… I become very tired…	Previously, my family said what is the use [of attending the training]. You are wasting your time. I started [to attend the training] on my own [despite their refusal] and now it has benefited me a lot.<br><br>I: can you please explain that to me?<br><br>R: I have one uncle. He said it [the training] won't change your life why don't you leave it. I started to attend the training despite that.

C4				
C5	Attending training with children is difficult and the facilitators faced challenges during the training sessions. So, it is good to have other alternatives for the children. As I told you, if there are day care centres, they will bring your child to you at the end of the training. There is no one who can care for our child including our families due to their behaviour. Handling such kinds of children is difficult. I attended the training because I have another son at home who can collect him [my child with DD] from school …. let alone my neighbours, my mother is not willing to look after such kind of child… My [other] children are handling my child [with DD] because they have the awareness, but they will not live with me forever...		Time is very scarce. If you don't sacrifice for your child time is always very scarce….	
C6	It is difficult to bring your child. I have brought mine once to the training. The training took a long time. Naturally such kind of children get bored quickly and feel agitated.…I don't have any family [near me], my family are far away, we don't meet often. It is very difficult to give such big responsibility, I would say. At least, I am not willing to leave my child with anyone or I don't think they will understand her, she gets upset and angry, when I see this, I am not happy, I don't think it is possible….		From the training, parts that were difficult are those that were given to me to practise at home. First, most of the time she [my child] is with me around night time. During daytime, she is with a babysitter as I need to work, and the babysitter couldn't practise what I have learnt with her [my child]. It has become difficult for me…	
C7		One of the challenges is shortage of time and the other challenge is distance. If you come from a far place, transportation problem can be one of the challenges. I arrived at home around half past 8 or 9 at night after attending the training due to transportation problems. Other than these, there was no other problem and the training was very good.	Life is difficult…I don't have much time but when I am available at home, we [me and my child] practise what I have learnt...	I have only one sister…I only told her about the training. She was saying you are going to the training for no reason. I explained to her that after I received the training, she [my child] has stopped hitting people when I tell her not to do so…
C8	At the family level, I don't have anybody who can look after my child. It is only me. There is no one. If you ask me what about a baby sitter. I get scared. I am very scared [of leaving my child with a baby sitter]. Sometimes, I bother my family if I must go to somewhere. But I get worried….		…for me there was a problem. Most of the time when I go home to practise, I was not successful. I used to get very sad during the first two weeks…I wasn't successful because of the problem my child had, sometimes he will surprise you with what he is able to do but he doesn't sit still. He just had that problem. I am not talking about the current situation. He has changed now. At the beginning he just couldn't sit still. And I used to get frustrated….	
C9				
FO1	The training took a little more time than was depicted on the training manual which made parents to stay for longer time. This can be considered as a challenge. For some parents, since they don't have a place to put their children, they were obliged to bring them to the training and if the children disturb, they should be with their parents. But as there was a play room, a room	…parents may have work so when they come from work or if they need to do household chores, so might spend an hour or so on the road; to arrive from different parts of the city…	…The other is there should be a clear plan. The guideline states it like that; to have a clear plan on which day; at what time [to practise], for example, when the child arrives from school, for how many minutes to sit with him/her and practise; to do something with	

	dedicated for the children, it was not that difficult. But sometimes, when the children were disturbed, and when we realized that the parents couldn't stay away from them, there were times they bring them to the training room. When that happened, and the children started running around in the training room, there might be destruction... What I think could be done sustainably is taking care of the children in a day-care if there is an access. But I don't think these day care centres might be there everywhere. Even if there are, I doubt if they are fit for children with developmental disorder. Even if there is one in one of the eastern corners of the city, it is difficult and tedious for the parents to drop the child there and get back for the training. It is not that accessible. If it was to be done in this hospital, it might be difficult to hire someone to handle and take care of the children for the training as there might not be enough resource. So, it is difficult to find a solution for that.		objects or things like that, i.e., having a clear idea on what to do. The plan, as to when, how, at what time and how many days per week [to practise] requires some support. It needs reminding every now and then. Constant reminder is needed for issues that were discussed earlier to be continuously used. When preparing the activity plan, there should be clear and specific timing, frequency, and at what time interval things should be done must be recapped. Maybe they [caregivers] didn't realize its usefulness.	
FO2	The second thing is there were parents who bring their children [to the training] especially in the first session ….eh…. especially in the first session before we assigned people to care for them it created a bit of disturbance. This is because half of the attention of the parents might be on their kids. There was an interruption and disturbance. But later, most of them were taken care of. But still some of the kids tended to refuse [to be away from their caregivers]. But this thing could be arranged better for the sake of avoiding such kind of interruption and disturbance. This may be something that should be considered for next sessions…one kid was not willing to depart from his mom for the last three sessions. He was with us [in the training room] the whole time but fortunately he wasn't that much disruptive. He didn't disturb that much but still we weren't able to engage him in our playroom….as a result what we can do is for next time we may need to find and adjust the time for the parents. Like if they [the children with DD] have school in the morning there could be a system where we can use that time. Adjusting the time because I have told you some of them even after they say okay after we prepare the place and person despite that they [the children] are not willing. So, we should use that as an option. In the second place for those who have such kind of problem may be if the moms could come early and stay with them [the children] at the setup and after a while they would leave. If that is possible, I think we can use such kind of methods.			
FO3			Time is the first reason [for not implementing what parents have learnt at home]. That is because these parents spend their day at work. But when we think about it, since it needs just five minutes although they were told to try it with their children within a short period of time, the children have no patience	

			to try it repeatedly. Above everything patience is important. There was a routine that they are used to. This is different. In the training they were told to be patient and to continue practising the things with their children. It was difficult for them to do that. They said things such as “he [my child] refused and run away, he doesn’t listen to what I say.” There were such difficulties…	
FO4				… [when we do the first home visit] there was this house where the husband is at work and I guess it is the husbands’ brother that was telling her [the mother] not to come for the training. When we went there, we explained the training to her thoroughly and left our telephone number in case her husband’s brother has a question, she was never absent from that day on….
FO5	In my view, it is better if they don’t bring their children to the training. Firstly, although the children stayed at the children playing room, the parents were thinking about their children. I think it is better if the parents assign someone in their house to look after their children and if the children stay at their home…for example, the training room and the playing room were next to each other, those who brought their child to the training put their child at the playing room. If the door is knocked or if they [the children] screamed, these parents’ attention will be with their children. But if the child was at home, that wouldn’t happen…			One thing I have observed is that one of the caregivers’ husband said to his wife ‘do not waste your time on something you are not going to bring about change’. She was arguing with him very much saying that what about the benefits I get from it [the training] …

Supplementary material:

Theme 3: Challenges and barriers (part 2)

Sub-themes:

- Programme delivery challenges
  - Practical
    - Lack of preparation
    - Length and timing of training
    - Problems in relation to training materials
    - Practical challenges in relation to home visits
  - Other
    - Group dynamics (differences among participating caregivers)
    - Difficulty understanding the importance of playing with a child and praising a child
    - Difficulty with changing ways of disciplining
    - Expectation

Full quotes from all participants relating to theme 3 – programme delivery challenges are presented in the matrix below. Quotes included in the main manuscript text are highlighted in yellow.

**Challenges and barriers**								
**Programme delivery challenges**								
**Practical**					**Other challenges**			
	**Lack of preparation**	**Length & timing of training**	**Problems in relation to training materials**	**Practical challenges in relation to home visits**	**Group dynamics**	**Difficulty understanding the importance of playing with a child and praising a child**	**Difficulty with changing ways of discipling**	**Expectation**
1	…the person who came here to teach us needs to review the materials two or three days earlier. When we were very eager to learn, and he just reads [the manual] for us, that will be a gap and I may miss something in the meantime. So, it would be better if this thing could be improved.							
2								…We still want you [the programme team] to be by our side even now [after the training finished] …eh... I have said it in the beginning ... eh... It can be in *Yekatit* Hospital and in *Tikur Anbessa*, we have asked the organisation

								which provided the training to do a lot for us with our children by our side. All the parents think that we should not go our separate ways after this ...eh... We told this to the doctor at the end and she told us they would discuss about it ... eh... We had a very good time. We say continue supporting us and it should continue ...
3								
4								
5								
6		…there were parents who bring their child to the training. It is difficult to bring your child. I have brought mine once to the training. The training took a long time. Naturally such kind of children get bored quickly and feel agitated. More importantly, the trainers should look for a solution for this…						
7								
8								I am telling you the truth. I was very happy while I was taking the lessons. I felt like my life was changing and I felt like I was leading another life. Last time when we were told that only one session is remaining, honestly something inside of me dropped. I was startled and very sad. I cried when I was alone… I like what has

								started. It is very good. I wish it didn't end.
9		…the training time was not good for me…There is no one who can take her home [from school] and I told the school about this… I told the school I have no one who can look after her [my child] during my training times. Due to this, they gave me permission and she was absent from school every Tuesday and she was going to the training with me.						Because I was very eager, I expected more, more things than this…eh… I expected more than this…what was in my mind is as a mother you would be eager …eh… if it is done more than this, if they are made to be more independent …eh…if the training is not finished. That's it. I worry about her future…eh… I worry about that.<br><br>…what is going to happen next? We, parents came together and have raised this concern to Dr x [programme facilitator] …
D1		The time the programme was taking was beyond our expectation. Usually we started on time, especially the first section was long. First, when they reported their homework, we needed to patiently listen to them and give chance to all of them. This took time. Sometimes we needed to explain some issues and the questions they had. These things took a long time and as a result the training was becoming very	…regarding demonstration, I think that there was a lack of preparation in advance from our side. The trainers should rehearse the role plays of the demonstration. So, the trainers should meet in advance to rehearse. There were problems of referring from the book [training manual] in the middle of the training, which interrupts the training for some time. Acting the role play by reading from a book and acting out naturally is different in		The other challenge was that the team was a mixed group. It means, some of them are more educated and have better understanding. For others, some of the issues may not be easily understandable and clear… For example, when they were paired to do a role play, one of them acting as a parent and the other as a child, they were not able to understand the instruction given to them which might be related to their level of understanding. This might indicate that they	…The parents might have used the techniques we taught them, but they don't report it. Why they don't report it might be I think because they didn't understand how important it is; because mostly when we ask them, they say that they did it. The other is, in case of one or two families, they say that they gave the child a ball to go out and play. We explain to them that this is not the aim; they as a parent should play with him/her. Therefore, I think if it is this much difficult to explain the		…But I think we cannot fulfil all their expectations. At first, when the training started, we have explained the main aim of the training to them, so they understand what they are going to learn from each session. I am not sure but, some things might be difficult specially when some parents say that their children are making progress after they started applying what they have learned; for others when there was no progress and when it gets difficult, they may expect additional special

		exhausting and long…as it becomes long it is also difficult to keep their [the caregivers’] attention.	terms of the distraction it creates on the viewers; and acting from inside depends on the preparedness of the trainer. So, what I think is that, when we were looking up and down checking the book continuously during the demonstrations, it might have created distraction and might have also made the trainees not to fully focus on the training…. I think it seems that the role play was a little difficult for them at the demonstration session. They have two role plays. It might be difficult for them because of two reasons. One, as role play is educational in a way that a parent acts both as a parent and as a child which may not be that much comfortable to them. Second, during rehearsal, if our preparation is not good, when it keeps being interrupted, it may be difficult for them to be attentive and follow effectively...		might need more support than expected…<br><br>…there were two men and seven women. The men do talk but they were only two. There were also women who talk and participate well, who even want to interrupt to forward their opinion. There were also people who were shy. There were also one or two people who talk only when their name is called. We observe that they are uncomfortable. The other is, Amharic might be their second language. So, it may be difficult for them to communicate and may lack the confidence to explain the situation in Amharic. Since they can talk in Amharic, it is difficult to identify their problem because they can talk and explain but they may not understand easily and quickly. When stories are read or when demonstrations are in play, it may be very difficult for them to understand. Those people who you obviously can understand that Amharic is not their first language while they talk, may be shy in conversations as they will not be comfortable talking. So, for them it is not only the educational level but also the language, the	need for the parent to play with their child in urban areas where there is better awareness, I guess it would be more difficult in rural areas.<br><br>There is nothing that contradicts with the culture or offends people. However, there is an issue of not understanding the importance of playing with their child… when they sit down with their child and play with toys, their neighbours may make fun of them and may say ‘are you going back to be a child again?’ In fact, none of the parents said this has happened to them but it can be anticipated. A parent may not be comfortable when others see him/her playing with toys with a child. Things like these might make them reluctant…		support. There are also parents who asked as what we can do to help them do such things…

					culture and gender have its own influence…			
D2	maybe if the material gets ready earlier because first, after the translation of the material we may not have enough time [to get ready]. Second, sometimes people working here have other responsibilities, so you may need to give them enough time to prepare, you need to read….eh…. you need to prepare…eh…as a result it is better if it is made available earlier. And I think that it is always useful to compare it with the original version.	…Another difficulty is sometimes because the sessions were conducted in the afternoon sometimes there was fatigue. There were situations where you just see plain exhaustion… The other thing is for me personally sometimes the time is too long. There is a tendency for the sessions to be boring and sometimes the sessions may last until the evening……eh…. there are people whose homes are far, there are parents who pick their kids up from another place. When you see it from that perspective there are times where the session is too long….eh….so how can we make it shorter? If there is a redundancy, sometimes if there is redundancy reducing those repetitions and removing parts that are not an absolute must. It would be good if such kind of things are done….	…. eh…eh…. the other thing is on the demonstration part there is something that requires us to act and there is also a message that we tell the caregivers there are number of interruptions. As a result, if it is not repeatedly read or it might be difficult to figure out the steps, where should it start? When should we start the act? It might be difficult to run it smoothly. Otherwise there might be a need to practice more……eh…. maybe if the part where we tell them the message would be at the beginning and at the end. If the people could clearly see what we are trying to say. When the acting and the message gets mixed up it creates a situation where it becomes difficult to clearly understand and get a clear message….		…The caregivers that were present are at different levels. Half of them are very fast, they are literate, they know a lot, they read a lot but a certain number of them have problems with reading and it may be difficult for them to understand things. Hence due to their understanding, there might be situations where they may not immediately equally understand everything. So, there might be differences and misunderstandings around that area…. would it be better if their composition is varied or similar? it may be in terms of educational level or it may be in terms of their child's problem and like sometimes they talk different things. Some of them have a very severely impaired child and some of them have very active kids. The things that gives sense to one might not give much sense to the other. As a result, maybe when we select them, we may need to make them a more similar group of people. Maybe if we give it to different group of people… I don't know maybe on the exclusion criteria if there are things		There was no content that offended them [caregivers]. However, there was a shift from the traditional caregiving or in terms of approach… the way they [traditionally] do things is if a child is refusing [to do something] or if he is disturbing, they yell at him or punish him. That part [practice] is still there with them. However, in our teaching when we teach them that punishment doesn't work and it's not that helpful…that may be a new change for them… I think ultimately over time they will understand it.	

					that we should watch out for. We may need to revise it again. So, we should concentrate on that….			
D3	My concern about the future is…. yes…each item in the manual should be read ahead of the training. It shouldn't be read roughly thinking that you understand the material. …eh…Each thing should be read and elaborated before delivering the training. …eh…It is not about reducing the time it takes to deliver [the training]. But the lead facilitator should understand the concept well and should explain to the trainees clearly. Otherwise it will be missed. The assistant facilitators were also good. But before they perform the role plays, they should have more preparation…eh... they should understand it well…eh… otherwise the parents are going to understand what they saw…eh…they are going to apply what they saw there…eh…Therefore, I think both the lead facilitator and assistant facilitators should understand the topics well and be prepared…	… the time allocated, and the material was not balanced. It was relatively vast. Relative to the time allocated it was vast. Thus, the facilitators had to rush up and cover all the material. The time allocated was not sufficient to give enough time for the parents to have a discussion. Thus, if that could be considered and if it can be revised and summarized. Otherwise the nine sessions were adequate. However, the last sessions were vast. I suggest if such sessions could be summarised….		…The minor challenge regarding home visits was that we couldn't arrive at their house on time. That was because there was a difficulty in finding their right house address…				
D4				…we didn't encounter any problem while doing the home visits. We wanted to see how they make their children play, how	I see the mothers as three types. As I told you, on the home visits, I saw hopeless mothers, I saw mothers in the middle hoping to see a better day and I have			

				they played together…	come across those who are really determined. I have worked before at a community level. I have not come across mothers like this, they are so determined to see their children achieve all that they can even with the problems that they have. They work on their knowledge, on related matters by asking, reading or making other children browse the internet to help their child reach his destination…. Mothers who are hurt a lot come to the training, but they keep quiet, I have seen one or two mothers who are always quiet and don’t answer our questions. They don’t participate or say only a few words in the group discussions. It is because of what they have experienced due to the hardships of having a child with autism and the fact that nobody understands them, and they do everything themselves for this reason and others they choose to keep quiet rather than talking about it. When I am facilitating, I try to make them participate, how are we going to approach such mothers? People get relief when they talk about their problems. It eases the tension for			

					them. I tried to make them participate. I have a little hesitation on this matter other than that everything was good, they had a good time…			
D5	…What, I think, needs to be improved is that the material was not ready on time…Initially the material and the translation were not given to us early. That was a problem. That was a setback for us to get enough time to get prepared well…	I: …eh the second thing is the time allocated and the time it [the training] took was not balanced. That is what I think should be improved…I mean the materials for example it was allocated for two hours and fifteen minute or two hours and half and so on. But it was taking more than three hours and the like. That had brought some problems to the participants.			As an idea, all of them have accepted the lessons, all the nine [sessions] properly. eh however because of time constraints, sometimes since we had to finish it, to finish the lesson, we had to go through it faster. During that time, if we look at them as a student, they have different levels of understanding. There is higher level, medium and lower level. Thus, there were three types of students in that class. There were such things within the training. Thus, because of time constraint there was no extra time to explain things to those who didn't understand it well.			

Supplementary material:

Theme 4: Suggestions for improvement

Full quotes from all participants relating to theme 4 are presented in the matrix below.

	**Add topics and feedback on how a session or sessions went**	**Adjust training time and length of training by simplifying/reducing some training materials**	**Continue to support caregivers/children after the programme completed**	**Programme facilitators prepare ahead of time**	**Suggestions on participants' composition/sitting arrangement**
C1		…it would be better if five or ten parents could attend the training together for certain days [shorten the number of days the training is being given] …		…the person who came here to teach us needs to review the materials two or three days earlier.	
C2			…We still want you [the CST team] to be by our side even now [after the training finished] …eh... I have said it in the beginning ... eh... It can be in *Yekatit* Hospital and in *Tikur Anbessa*…All the parents think that we should not go our separate ways after this ...eh... We say continue supporting us…		
C3			…It will be good if there is some financial support. Me, for example, I need to pay house rent. I live with lots of difficulties. His [my child's] care needs lots [of money]. That is because I can't work when he is sick. There are times that I work for only two days [per week] and then do not go to work. I need to take him to a health facility. For that reason, it is so difficult. I don't work enough, and my husband is also working as a daily labourer…		
C4					
C5	…how to use potty and toilet, how we should teach [our children] this. This is a big thing. The other thing is some children can't eat by themselves and they depend on their mothers. If the training includes what mothers can do for such kinds of children, it will be good. Otherwise the training was very good…		The training is good, but these children will be a burden for the community and the country if they don't get knowledge…it would be good if something could be arranged for these children to learn…if there is a centre for these children…		
C6	…with these children, there is a need to be extra careful to avoid				

	rape or other kinds of abuse from happening. There are many things that we see be it at home [or outside home] that should be included in the training, in what ways should we prevent such abuses from happening, if these ideas could be included in the training it would be good.				
C7	…what I want to suggest for the future is that we should train these children how to protect themselves because they are growing…For example, my daughter is not scared of anybody and follows other people to places. She may go with other people to far places and she may not be able to come back home. It would be good if I could get lesson on how to explain this [the danger of following strangers] to her….				
C8			…I had enrolled him [my son] to a school and I was happy. Because he was learning, and he was going out of the house…But because they [the school] were unable to handle him, they told me to take him out. After he stopped school, I was very sad. I wish they [children with DD] have their own school. If they could go out of the house and stuff. Seeing that would make me happy. I was very happy when he went to school. And he even got used to it. [After he stopped school] he used to get up in the morning and pick up his bag and uniform [thinking that he is going to school]. It took some effort to make him stop doing that….		
C9			…what was in my mind is as a mother you would be eager …eh… if it is done more than this, if they [the children with DD] are made to be more independent …eh…if the training is not finished. That's it. I worry about her future…eh… I worry about that.		

FO1				…the demonstration should be rehearsed by the facilitator and the co-facilitator in advance. We usually try to rehearse for the role play at 1:30pm or 1:40pm, when we arrive early, and the classes are empty before the training program starts at 2:00pm; using these 20 minutes; or on the break, for the role play to be played after the break. But I don't believe that this is enough.	…As for the mixed group, it could be better to make it uniform. As per the feedback I got from the trainers, when they talk to each other some of the trainees do not understand easily. So, when the training is repeatedly given to the ones who don't understand, the ones who do, might get bored. So, it is better to try to make the team homogeneous. But on the other hand, when it becomes homogeneous, it decreases the learning process with each other. The fact that it was heterogeneous group creates an opportunity for discussion and a chance to learn from each other. But had the team been homogeneous the training could have been given quickly and actively. For those who do not understand easily, it could be better to give them the training taking time using examples and leaving out all the challenging parts from the training.
FO2	…may be some additional tips in order to engage some parents; there are those parents with children with additional problems, that is in addition to the delay there is a tendency to be hyperactive, to be restless. From that perspective if there could be tips at least for the facilitators on the correct way to address this….<br><br>…after the session, summarizing the session and sort of giving feedback… so it may be at the end of the session or by taking time on some other day; [saying] this was good, this was bad throughout the session, these kinds of things are good for improvement. It shouldn't be when every session was completed…. But we can have another program or before starting the next session	…Like if they [the children with DD] have school in the morning there could be a system where we can use that time. Adjusting the time because I have told you some of them [caregivers] even after they say okay after we prepare the place and person despite that they [the children] are not willing. So, we should use that as an option….<br><br>…there are times where the session is too long….eh….so how can we make it shorter? If there is a redundancy, sometimes if there is redundancy reducing those repetitions and removing parts that are not an absolute must. It would be good if such kind of things are done…		…. eh…eh…. the other thing is on the demonstration part there is something that requires us to act and there is also a message that we tell the caregivers there are number of interruptions. As a result, if it is not repeatedly read or it might be difficult to figure out the steps, where should it start? When should we start the act? It might be difficult to run it smoothly. Otherwise there might be a need to practice more…	

	we can assess how well things were going or what things weren't going well. [We can ask] where was the problem? This is good so that in the process things will get modified… Be it on the method of delivery if there is no proper feedback the problems may last until the end. So, there is a need to revise.				
FO3		… the time allocated, and the material was not balanced. It was relatively vast. Relative to the time allocated it was vast. Thus, the facilitators had to rush up and cover all the material. The time allocated was not sufficient to give enough time for the parents to have a discussion. Thus, if that could be considered and if it can be revised and summarized. Otherwise the nine sessions were adequate. However, the last sessions were vast. I suggest if such sessions could be summarised….			
FO4					…I have asked a question there [during the training sessions] on mothers sitting arrangement. I thought it would be best if they exchange places after one or two sessions. They stay at a particular place for all the nine weeks and by exchanging places they could exchange their experiences… I think it would be helpful for the mothers to sit with different individuals at different weeks. This will help them, other than this there is nothing I would suggest…
FO5			…there is nothing I would say should be added. But after the training completed, if caregivers could meet up. For example, in stead of immediately stopping to meet up once a week, maybe meeting up once a month or three months to see their progress. To see their progress after		

			the training. If we could arrange this, I think, it would be good…		
